# Gene expression signatures in PCB-exposed Slovak children in relation to their environmental exposures and socio-physical characteristics

**DOI:** 10.1007/s11356-022-20018-2

**Published:** 2022-04-14

**Authors:** Tanmoy Mondal, Christopher A. Loffredo, Tomas Trnovec, Lubica Palkovicova Murinova, Zarish Noreen, Thomas Nnanabu, Kamil Conka, Beata Drobna, Somiranjan Ghosh

**Affiliations:** 1 Department of Biotechnology, Maulana Abul Kalam Azad University of Technology, Salt Lake, Kolkata, 700064 India; 2 Department of Oncology, Georgetown University, Washington, DC 20057 USA; 3 Department of Environmental Medicine, Faculty of Public Health, Slovak Medical University, Bratislava, Slovak Republic; 4 Department of Healthcare Biotechnology, National University of Sciences and Technology (NUST), Islamabad, 44000 Pakistan; 5 Departments of Biology, Howard University, 415 College Street, NW, Room 408, EE Just Hall, Washington, DC 20059 USA; 6 Department of Toxic Organic Pollutants, Faculty of Medicine, Slovak Medical University, Bratislava, Slovak Republic; 7 Departments of Pediatrics and Child Health, College of Medicine, Howard University, Washington, DC 20059 USA

**Keywords:** PCBs, Gene expression, Biomarkers, Organochlorines

## Abstract

Our previous gene expression studies in a PCB-exposed cohort of young children in Slovakia revealed that early-life exposures to PCBs and other organochlorine compounds were associated with significant alterations across several pathogenetic pathways. The present study was undertaken to further explore the high-throughput qRT-PCR-based gene expression effects by using TaqMan low-density array (TLDA) for selected genes in a sample of 55 children from the cohort. We analyzed the transcriptional changes of 11 genes in relation to PCB and organochlorine pesticide exposure levels (including DDT, DDE, HCH, and HCB), and to BMI and ethnicity in this cohort. The results indicated an overall downregulation of expression of these genes. Maximum downregulation (in fold change) was observed in the *ENTPD3* gene, and the minimum level of downregulation was in *CYP2D6*. As per our multinomial regression model study, downregulation of *LEPR* gene was significantly directly correlated with all the exposure variables. Downregulation of *APC*, *ARNT*, *CYP2D6*, *LEPR*, *LRP12*, and MYC genes was directly correlated with BMI (kg/m^2^) of the individuals. Gender-specific differences in gene expression were observed in *CYP2D6* (*p*-value 0.0001) and *LEPR* (*p*-value 0.028), while downregulation of *CYP2D6* (*p*-value 0.01), *LEPR* (*p*-value 0.02), *LRP12* (*p*-value 0.04), and *MYC* (*p*-value 0.02) genes was consistently observed in Roma children compared to Caucasians. The investigation of such health disparities must be emphasized in future research, together with interventions to reduce the health consequences of PCB exposures. In this context, we emphasize the importance of biomarker-based approaches to future research on genetic susceptibility to the effects of these compounds.

## Introduction

Polychlorinated biphenyls (PCBs) are one of the most persistent environmental chemical toxicants that has been recognized by the United States Environmental Protection Agency (USEPA), The Agency for Toxic Substances and Disease Registry (ATSDR), World Health Organization (WHO), and The International Joint Commission (IJC) (USEPA, 2003; ATSDR, 2000; WHO, Food Safety; Chemical Risk in Food 2005; IJC, 2002). Due to their stability, PCBs are very persistent in the environment. Despite a ban on their production since 1979 (in the USA), body burdens of PCBs continue to accumulate in humans owing to the dumping of these compounds (Sun et al., 2007; Hsu et al., 2003). Due to its lipophilicity, the body burden of PCBs further depends on adiposity and the dietary intake of high-fat foods (Smeds and Saukko, 2001; Covaci et al., 2002; Yu et al. 2007). Thus, the legacy of environmental PCBs is truly multi-dimensional.

Due to their structural differences, the modes of action of different PCB congeners can result in different disease outcomes (Carpenter, 2006). The structure–activity relationships studies suggested that coplanar PCBs have biological activities alike dioxin (2,3,7,8-tetrachlorobibenzo-*p*-dioxin) through the aryl hydrocarbon receptor activity, hence a potent carcinogenic compound (Carpenter, 2006; Safe, 1994). On the other hand, the non-coplanar PCBs (e.g., 99, 138, 153, 180, and 19) show more complex patterns of toxicity, having estrogenic and neurotoxic activities (Saint-Amou et al. 2006). Both coplanar and non-coplanar PCB congeners have been detected in human tissues and in the circulation; however, the nonplanar PCBs are the most prevalent and persistent congeners in the environment (Humphrey et al., 2000).

PCBs are often categorized as organochlorines, along with organochlorine pesticides (OCPs), which in turn are the most abundant of the persistent organic pollutants in the environment. They have been extensively used in agriculture as well as in public health measure (e.g., malaria eradication) worldwide, for the over several decades, and are even used in several developing countries, whereas they are banned in most developed countries (Jayaraj et al., 2016). These pesticides are also well-known endocrine disruptors (Mnif et al., 2011). OCPs such as dichlorodiphenyltrichloroethane (DDT), hexachlorocyclohexane (HCH), and hexachlorobenzene (HCB) have been found to be carcinogenic in some studies. Based on the aforesaid information, International Agency for Research on Cancer (IARC, 2014) categorized them as “possibly carcinogenic to humans.”

PCBs have also been associated and responsible for serious chronic diseases and disorders, viz., harmful reproductive health effects (Plísková et al., 2005), neurological deficits (Park et al., 2009), endocrine effects (Rádiková et al., 2008), hearing losses (Trnovec et al. 2008), including diabetes, cardiovascular diseases, and cancers (Ghosh et al., 2014; 2015; 2018). Developmental effects from exposures to PCB congeners have also been reported (Royland et al., 2008).

In eastern Slovakia, improper disposal of PCBs over several decades caused an extended period of contamination of freshwater sediments (Kocan et al., 2001; Park et al., 2007; Wimmerová et al., 2015). Studies between 1987 and 1990 in Slovakia observed elevated concentrations of PCBs in food (Hertzman, 1995). A study from the breastfeeding mothers of Michalovce district also showed that concentrations of the PCBs in breast milk averaged from 4.0 to 4.4 mg/kg lipids (Hertzman, 1995), which greatly exceeds regulatory safety levels (< 0.01–0.04 ng/g) (Korrick and Altshul 1998). The typical PCB concentration (the sum of PCB — 28, 52, 101, 138, 153, 156 170, and 180) in human blood lipids in overall population living long term in the Michalovce District (highly contaminated) was 3.5 times higher than Stropkov District (with lower exposure only). The serological analysis revealed that PCB 153 and PCB 138 are the prevalent congeners, comparable to other studies during that time frame (Ghosh et al., 2009; Hovander et al., 2006; Petrik et al., 2006; Jursa et al. 2006). Population-based investigation in this area has also shown deleterious effects in neuro-behavioral development, and reduction in thymus size at birth (Park et al., 2008; Šovčíková et al. 2015).

To date, gene expression studies on the Slovak cohort (Ghosh et al., 2015; 2018) have showed relationships of PCB exposures with alterations in the expression levels of multiple genes in the developing disease and disorder (in pathways) that are in accord with other studies carried out by us (Ghosh et al., 2013; 2014; 2015; Mitra et al., 2012). Those prior findings suggested that certain genes were downregulated at elevated PCB exposure concentrations. However, dependency or any correlation of the gene downregulation with associated factors like DDT, DDE, HCB, or HCH co-exposures, and individual characteristics such as gender, BMI, and ethnicity were not studied in detail. The aim of the present investigation, therefore, is to address these knowledge gaps by further characterizing the expression levels of a panel of candidate genes that emerged from our prior work, in relation to PCB and OCP exposure levels and personal characteristics of children in this unique cohort.

## Methods

### Analysis of PCBs/POPs

Analyses of 15 PCB congeners (e.g., PCBs 28, 52, 101, 105, 138, 114, 118, 123 ^+^^149^, 153, 156^+^^171^, 157, 167, 170, 180, and 189) and also p,p'-DDT, p,p'-DDE, HCB, and HCH (α, β, and γ) in the serum was done using gas chromatography (High-Resolution; 6890N; Agilent Technologies, Santa Clara, CA, USA) coupled with a Ni-63 micro-electron capture detector and a 60-m DB-5 capillary column (J&W Scientific, Folsom, CA, USA) (Kocan et al., 1994; 2001; Conka et al. 2005; Petrik et al., 2006).

### Study participants

Howard University Institutional Review Board (IRB-07-GSAS-30) and Ethics Committee of the Slovak Medical University in Bratislava (Dated April 2006) approved the study. Participants included in this study were among cohort of mother–child pairs, in the study “PCBs and Early Child Development in Slovakia”, reqcruited between 2002 and 2004 (Hertz-Picciotto et al., 2003; Sonneborn et al., 2008a, b). The enrollment and description of this cohort can be found in details in Ghosh et al. (2018). We selected 71 participants (boys = 30, girls = 41) from our earlier study (Ghosh et al., 2018) and built upon their blood PCB measurements at the age of 45 months, aiming to compare and contrast the low- and high-exposure subsets. Out of the 71 participants, 55 (boys = 25, girls = 30) were included in the present study and the rest of the 16 participants were excluded due to incomplete data set. Regarding the ethnicity of the population, 13 were Roma (Gipsy) and 42 were Caucasian. The gender distribution, ethnicity, and other details of the participants are summarized in Table 1. There are no substantial variations in body weight and BMI between boys and girls and between Roma and Caucasian group in our study population.

**Table 1:** The distributions of gender, ethnicity, and other characteristics of the participants

	Overall	Male/female	Ethnicity (Roma/Caucasian)
Number of participations	55	25/30	13/42
Body weight (kg)	16.83 ± 2.81	16.52 ± 1.10/17.06 ± 3.33 (*p*-value 0.49)	15.83 ± 2.21/17.12 ± 2.93 (*p*-value 0.16)
Height (cm)	95.96 ± 6.79	96.34 ± 7.90/95.66 ± 5.90 (*p*-value 0.72)	90.75 ± 6.29/97.49 ± 6.20 (*p*-value 0.001)
Breast feeding (months)	10.14 ± 9.91	10.12 ± 10.95/10.16 ± 9.13 (*p*-value 0.98)	17.15 ± 12.89/7.97 ± 7.76 (*p*-value 0.002)
Gestational age (weeks)	39.87 ± 0.80	39.88 ± 0.6/39.86 ± 0.95 (*p*-value 0.93)	40.15 ± 0.69/39.78 ± 0.82 (*p*-value 0.14)
BMI (kg/m^2^)	18.37 ± 3.13	17.99 ± 2.70/18.67 ± 3.44 (*p*-value 0.44)	19.22 ± 1.81/18.12 ± 3.40 (*p*-value 0.28)

Results are displayed are mean ± SEM

## Body mass index (BMI)

The body mass index (BMI) for all subjects was recorded at the medical clinic at the age of 45 months and was expressed in units of kg/m^2^ here by capturing their height and weight data during enrollment.

## Sample collection and RNA preparation

Blood samples from the children at the age of 45 months, with prior parental consent, were collected into a PAXgene™ blood RNA tube (IVD; BD Biosciences) by certified phlebotomist under the direction of the medical team from Slovak Medical University (Park et al., 2007). The samples were transported to Bratislava in a cold chain prior to shipping to collaborators’ lab at USA, through special air freight carrier. The RNAs were isolated by using PAXgene Blood RNA kit (Cat # 762,164, PreAnlytiX GmbH, Germany) and TRIzol® Plus RNA Purification System (Invitrogen, California; CA), respectively (Ghosh et al., 2015; 2018). The RNA was stored at − 80 °C if not worked on immediately (within 24 h).

## cDNA synthesis

High-capacity cDNA Reverse Transcription Kits (Part # 4,387,406; Applied Biosystems, CA, USA) was used for cDNA synthesis (see Ghosh et al., 2018 for detailed procedural description). The cDNAs were stored in − 15 to –25 °C, if not used immediately (within 24 h), or stored in 2 to 8 °C prior to downstream application.

## High-throughput TaqMan® low density array (TLDA)

Custom-made TLDA cards (Applied Biosystems, CA) were used to analyze the expression of eleven genes of importance (*APC*, *ARNT*, *BCL2*, *CD3G*, *CYP2D6*, *ENTPD3*, *LEPR*, *LRP12*, *MYC*, *RRAD*, and *TRAP1* from our prior research in this cohort (see details in Ghosh et al., 2015; 2018).

## TLDA data analysis

SDS Ver. 2.4 software (ABI, CA) was used for the TLDA data analysis. Threshold cycle (Ct) data for all focused genes and control gene 18 s RNA were used to determine the ΔCt values [ΔCt = Ct (target gene) − Ct (18 s RNA)]. The ΔΔCt values were then computed by deducting the calibrator (control) from the ΔCt values for each target(s) gene. DataAssist V2.0 (ABI, CA) allowed us to visualize the expression of each gene in the corresponding individuals.

## Statistical analysis

Student’s *t*-test was used for comparisons between two groups, including high- versus low-exposure status, and by gender and ethnicity. To determine the relationship between individual factors and gene expression, we performed linear regression analysis and checked the slope to find any observable linear association. Reported data are represented as means ± SEM. Linear regression analysis was performed using GraphPad Prism (version 8) software. The differences observed and the data with *p*-value < 0.05 were stated as statistically significant.

A multinomial regression model was applied (using SAS, version 9.4) on all 11 genes simultaneously, with adjustment for ethnicity, gender, BMI, and all exposure variables (PCBs, HCH, DDE, DDT, and HCB). All the variables that were not normally distributed were log-transformed prior to the analysis. Because of the significant inter-correlations between the exposure variables in the data set, we analyze one exposure variables at a time in relation to the gene expression levels and patient variables.

## Results

### Overall differential gene expression of 45-month old Slovak children

**Fig. 1:**
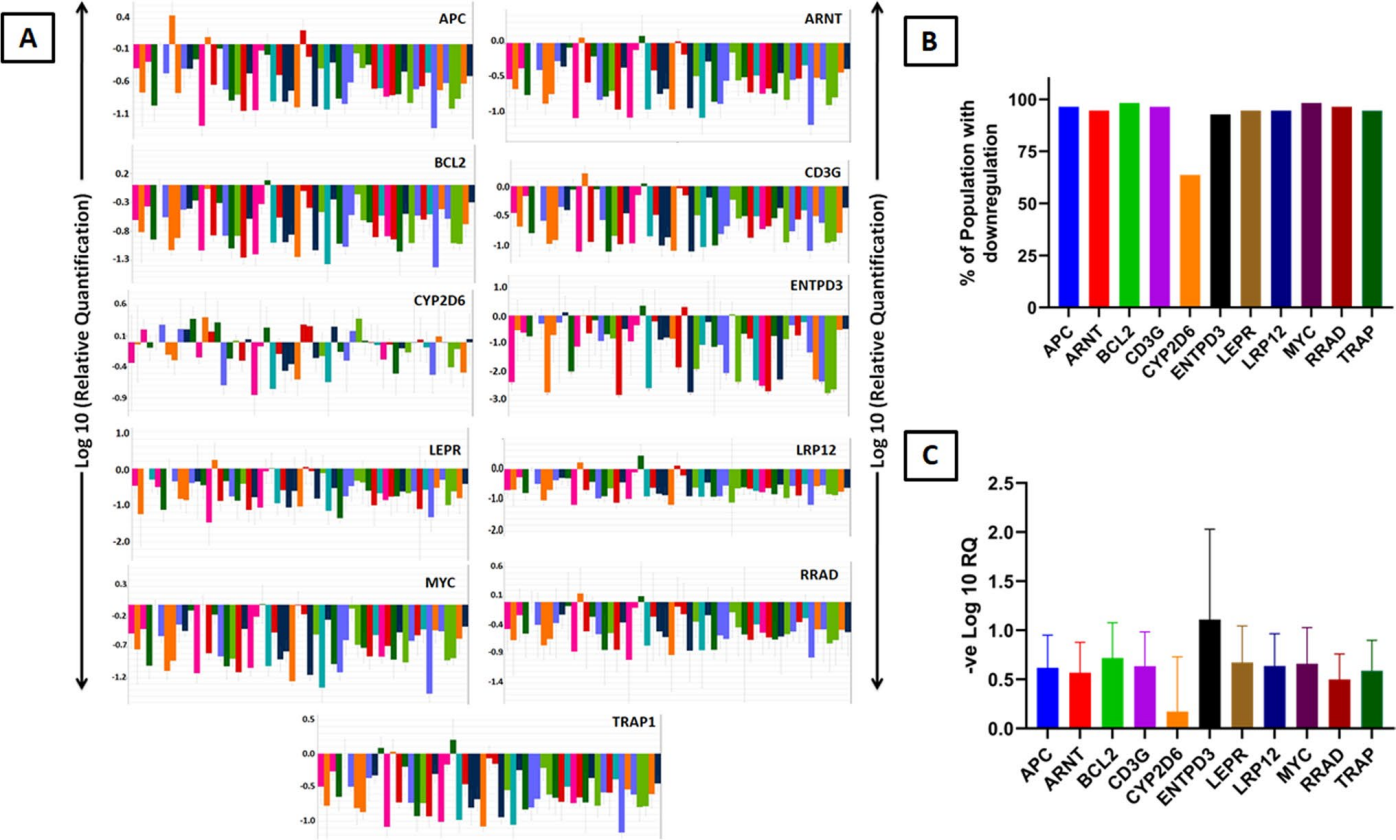
**A** Quantitative real-time PCR (qRT-PCR) validation of the selected 11 genes of interest by TaqMan low-density array (TLDA) in ABI platform (7900HT Fast Real-Time PCR System) after analyzed (ΔΔCt) by SDS RQ Manager Version 1.2.1. The relative quantification (RQ) of the genes showing up-/downregulation among the subjects in a small population (*n* = 55) validation. The RQ is calculated in contrast to calibrator samples, i.e., the subjects with no/minimum background PCB exposures in the population. **B** Histogram of total percentage of population (*n* = 55) having downregulation of individual genes. **C** Histogram of average fold change (downregulation) of individual genes in overall population

Out of the 11 candidate genes, we observed that the majority was differentially expressed (both up-/downregulated) (Fig. 1A): all ten genes were downregulated in 49 participants (89% of the population), except for the *CYP2D6* gene, which was downregulated in only 63.63% (*n* = 35) of the total population (Fig. 1B). Maximum downregulation (fold change) was observed for *ENTPD3* (− 1.21-fold change). Minimum level of expression was for *CYP2D6* gene (− 0.38-fold change) (Fig. 1C).

**Fig. 2:**
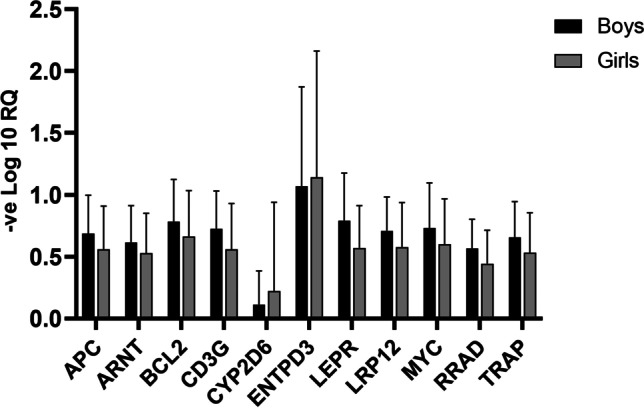
**A** Comparison of average fold change in terms of downregulation (− log 10 of relative quantification) of individual genes in between male and female group

A notable difference in the relative quantification of selected gene expression was observed in between boys and girls (Fig. 2), where most of the genes (except *CYP2D6* and *ENTPD3*) were more downregulated in the boys group compared to the girls group. Out of the 11 genes, a gender-specific significant change of gene expression observed in *CYP2D6* and *LEPR* gene with an observed *p*-value was 0.0001 and *0.028 respectively (Fig. 2).

## PCB exposure and gene expression

Blood samples were collected from the Michalovce District, which is considered high PCB-contaminated area, where PCBs 153, 138, 180, and 170 were the most abundant PCB congeners (Ghosh et al., 2018). The average concentrations of PCB 153, 138, 180, and 170 were 185.04 ± 193.92 ng/g lipid, 123.44 ± 135.20 ng/g lipid, 132.19 ± 141.73 ng/g lipid, and 52.98 ± 57.98 ng/g lipid respectively. We did not observe any significant correlation between PCB concentration (ng/g lipid) and individual gene expression.

## HCH exposure and gene expression

**Fig. 3:**
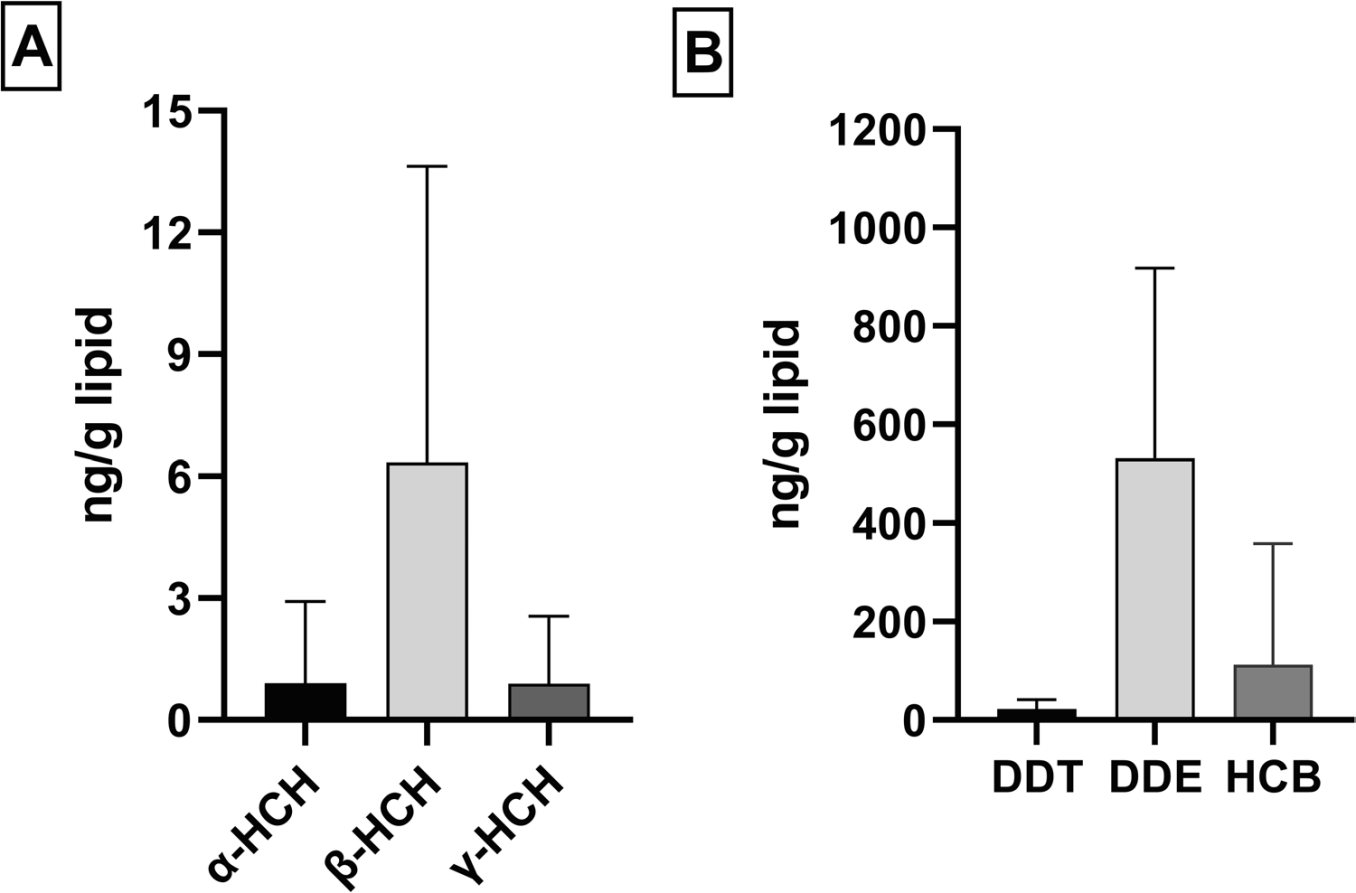
**A** Histogram represents average amount of HCH (α, β, and γ-HCH) present into the total population (*n* = 55). **B** Histogram represents average amount of DDE, DDT, and HCB present into the total population (*n* = 55)

The mean levels of α, β, and γ–HCH in the study population were 0.91 ± 2.01, 6.34 ± 7.29, and 0.89 ± 1.67 ng/g lipid, respectively (Fig. 3A). There were no statistically significant associations of HCH with gene expression although several marginal associations (p<0.07) were observed between γ–HCH and four different gene expression (*BCL2*, *CD3G*, *CYP2D6*, and *LRP12*).

## DDT, DDE, and HCB exposures in relation to gene expression

The mean levels of DDT, DDE, and HCB were 22.36 ± 81.71, 531.16 ± 385.93, and 112.30 ± 246.09 ng/g lipid, respectively (Fig. 3B). DDT, DDE, and HCB individual levels were not significantly associated with gene expression levels.

## Ethnicity and relative gene expression

**Fig. 4:**
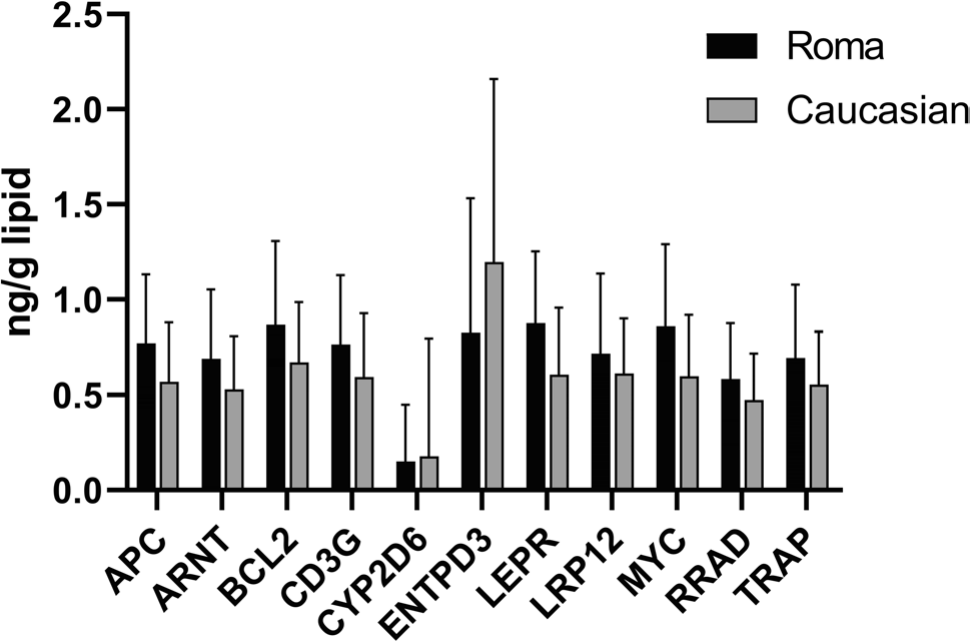
Comparison of average fold change after quantitative real-time PCR (qRT-PCR) validation of the selected 11 genes of interest in terms of downregulation (− log 10 of relative quantification) in between Roma and Caucasian group

Gene expression levels differed by ethnicity (Fig. 4). Out of the 11 genes, an ethnicity-specific substantial difference in gene expression was observed in *CYP2D6* (*p*-value 0.01) and *LEPR* (*p*-value 0.02), *LRP12* (*p*-value 0.04), and *MYC* (*p*-value 0.02).

## BMI and relative gene expression

**Fig. 5:**
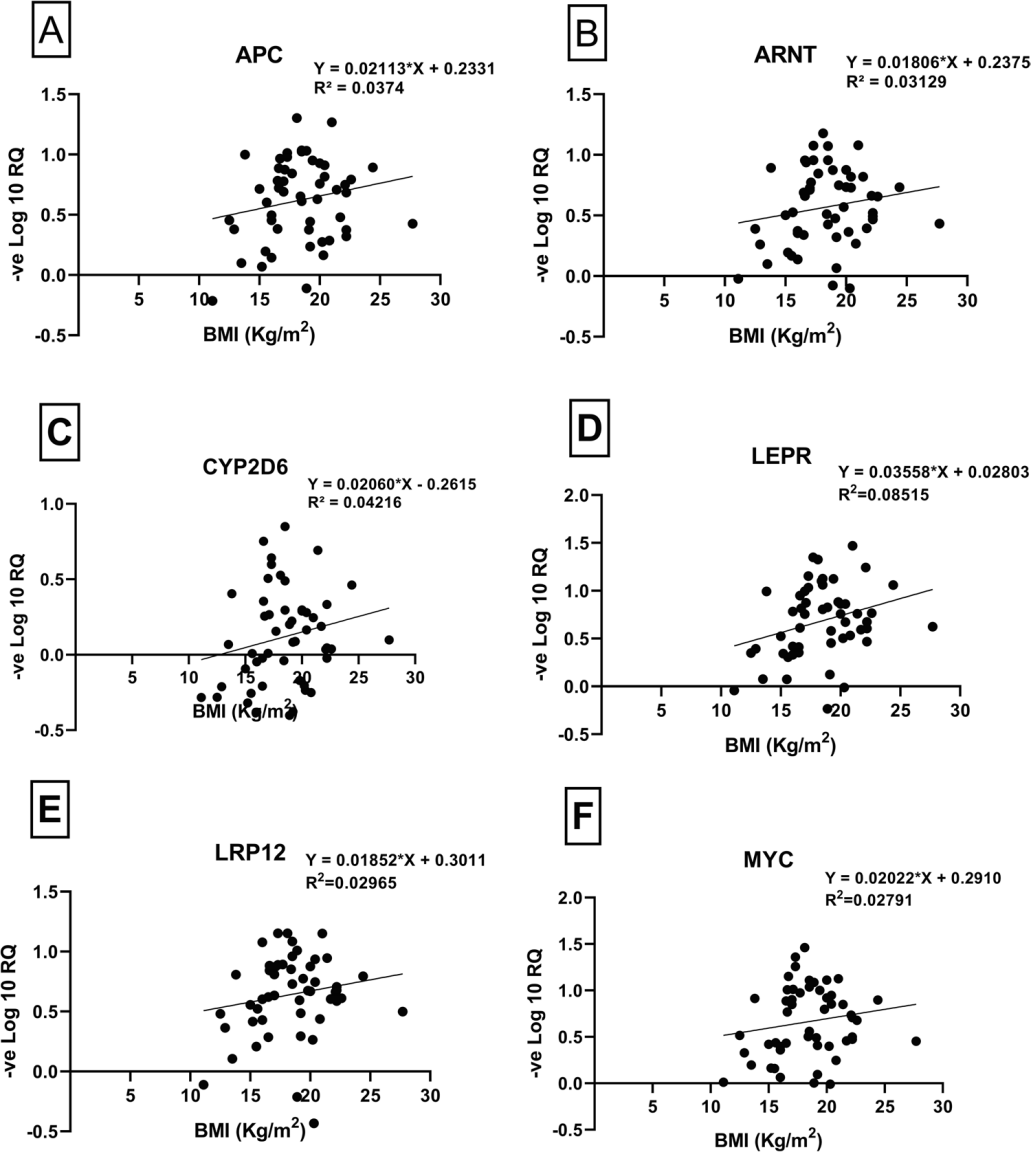
Regression plot analysis between BMI (kg/m^2^) of individual participants and differential expressions of selected six genes (**A** APC, **B** ARNT, **C** CYP2D6, **D** LEPR, **E** LRP12, and **F** MYC)

Out of 11 differentially expressed genes, lower levels of expression of *APC* (*R*^2^ = 0.0374), *ARNT* (*R*^2^ = 0.0313), *CYP2D6* (*R*^2^ = 0.0421), *LRP12* (*R*^2^ = 0.0296), and MYC (*R*^2^ = 0.0279) were correlated with increasing BMI of the individuals (Fig. 5A–F).

## Multinomial linear regression results

To assess the multiple effects of gender, ethnicity, BMI, PCBs, and other OC exposure levels on gene expression levels, we performed multinomial linear regression on the set of 11 genes. The results showed that the overall model (11 genes included) was not statistically significant (*p*-value 0.43) after adjusting for ethnicity, gender, BMI, and all exposure variables (sum of PCBs, α-HCH, β-HCH, γ-HCH, HCB, DDE, and DDT). We subsequently investigated the relationship of one exposure at a time with a single gene at a time, while adjusting for ethnicity, gender, and BMI. We observed a significant relationship between *LEPR* gene and with all the exposure variables, including sum of PCBs (*p*-value 0.0046), with α, β, and γ–HCH (*p*-value 0.0081, 0.0110, and 0.0045 respectively), DDT (*p*-value 0.0105), and DDE (*p*-value 0.0091). We also found a significant correlation between sum of PCBs and *MYC* (*p*-value 0.0449), γ–HCH and *MYC* (*p*-value 0.0596), and γ–HCH and *CYP2D6* (*p*-value 0.0377) genes using such models.

## Discussion

The current study was designed to explore the transcriptional profiling of a panel of 11 genes in PCB-exposed Slovak children using high-throughput qRT-PCR. The aim was to explore the effects of co-exposure to other persistent organochlorine exposures and the potential associations of the gene expression levels with gender, ethnicity, and BMI, which have never been reported for this cohort. The results confirm the prior findings of strong relationships between PCBs and a general pattern of downregulated gene expression. Other types of organochlorine exposures were not strongly related to gene expression levels. Increasing BMI was linearly associated with increases in expression levels of 6 of the 11 genes we profiled.

The 11 genes in this study, e.g., *APC*, *ARNT*, *BCL2*, *CD3G*, *CYP2D6*, *ENTPD3*, *LEPR*, *LRP12*, *MYC*, *RRAD*, and *TRAP1* demonstrated significant expression changes, which in turn have a foremost effects in facilitating toxicities by modifying cellular and molecular events towards development of disease and disorders, i.e., cell cycle, cellular movement, cell death, cancer, metabolic disorder, neurological diseases, tumor, genetic disorder, and immunological diseases as the most common, underlying functions (Table 2), previously validated in an in vitro transcriptional profiling study (Ghosh et al., 2015) and biological pathway analysis (Ghosh et al., 2018).

**Table 2:** List of selected 11 gene name, function, and percentage of population with transcriptional changes (both up ( +) and down ( −) regulated)

Sl No	Gene name (probe sets)	Description/functions	Population result (*n* = 55)
1	APC (Hs01568270_m1)	Adenomatous polyposis coli protein; WNT signaling pathway regulator	− 0.64 (*n* = 53, 96.36%) + 0.16 (*n* = 2, 3.63%)
2	ARNT (Hs01121918_m1)	Aryl hydrocarbon receptor nuclear translocator protein; it promotes the expression of genes involved in xenobiotic metabolism	− 0.60 (*n* = 52, 94.54%) + 0.06 (*n* = 3, 5.66%)
3	BCL2 (Hs99999018_m1)	B cell lymphoma 2 family regulator protein	− 0.73 (*n* = 54, 98.18%) + 0.08 (*n* = 1, 1.81%)
4	CD3G (Hs00173941_m1)	The genes encoding the epsilon, gamma and delta polypeptides. Defects in this gene are associated with T cell immunodeficiency	− 0.66 (*n* = 53, 96.36%) + 0.13 (*n* = 2, 3.63%)
5	CYP2D6 (Hs00164385_m1)	Cytochrome P450 family 2 subfamily D member 6 involved in drug metabolism and synthesis of cholesterol, steroids, and other lipids	− 0.38 (*n* = 35, 63.63%) + 0.20 (*n* = 20, 36.36%)
6	ENTPD3 (Hs00928977_m1)	Ectonucleoside triphosphate diphosphohydrolase 3; involved in the regulation of extracellular levels of ATP by hydrolysis of it and other nucleotides	− 1.21 (*n* = 51, 92.72%) + 0.20 (*n* = 4, 7.27%)
7	LEPR (Hs00174492_m1)	Leptin receptor; the protein is involved in the regulation of fat metabolism	− 0.71 (*n* = 52, 94.54%) + 0.09 (*n* = 3, 5.45%)
8	LRP12 (Hs00273787_m1)	LDL receptor–related protein 12; its related pathways are metabolism and metabolism of fat-soluble vitamins	− 0.69 (*n* = 52, 94.54%) + 0.25 (*n* = 3, 5.45%)
9	MYC (Hs00153408_m1)	MYC proto-oncogene, bHLH transcription factor; plays a role in cell cycle progression, apoptosis and cellular transformation. Amplification of this gene is frequently observed in numerous human cancers	− 0.67 (*n* = 54, 98.18%) + 0.008 (*n* = 1, 1.81%)
10	RRAD (Hs00188163_m1)	Ras-related glycolysis inhibitor and calcium channel regulator; diseases associated with RRAD include benign pleural mesothelioma and diabetes mellitus	− 0.52 (*n* = 53, 96.36%) + 0.12 (*n* = 2, 3.63%)
11	TRAP1-Hs00212476_m1	TNF receptor–associated protein 1; diseases associated with TRAP1 include Vacterl association and hereditary multiple exostoses	− 0.63 (*n* = 52, 94.54%) + 0.10 (*n* = 3, 5.45%)

As per our multinomial model results, the sum of PCBs and also α-HCH, β-HCH, γ-HCH, HCB, DDE, and DDT environmental exposures were significantly associated with the downregulation of the *LEPR* gene. *LEPR* gene was downregulated in most of the samples (94.54% of the study population), corroborating with our earlier investigation (Ghosh et al., 2013).

The *LEPR* gene works as a receptor for the fat cell-specific hormone leptin. It controls of fat metabolism and thereby regulates body weight, and it is also engaged in a distinctive hematopoietic pathway, important for normal lymphopoiesis (Bennett et al., 1996). The downregulation of *LEPR* (leptin receptor) gene disrupts the natural function of leptin. Equally, we suggest that the children previously have had the high pre- and postnatal exposure to PCBs could experience an alteration in the profile of leptin in early life. This may be linked with increased predisposition to obesity and metabolic disorders in adulthood. Earlier studies also reported that low dose exposure in young adults to p, p′-DDE (a persistent lipophilic metabolite of DDT), p, p′-DDT, and PCBs with more chlorine atoms predicted increased BMI in the future (Lee et al., 2011).

It is known that high BMI or obesity in childhood has significant impacts on both physical and psychological health (Smith et al., 2020). Overweight or obesity is now a worldwide health concern, where obese children will be most likely to remain obese rest of their lifetime. They are also more predisposed to have more non-communicable diseases like diabetes and cardiovascular diseases at much younger ages (Kelishadi and Heidari-Beni, 2019). BMI in our studied population showed a direct relationship with the transcriptional changes (downregulated) observed on selected genes (*APC*, *ARNT*, *CYP2D6*, *LEPR*, *LRP12*, and *MYC*) (Fig. 5), all of which are closely linked to the development of cancer or obesity (Ghosh et al., 2015).

The ethnicity in the studied population revealed an interesting relationship with the observed transcriptional patterns. Genetic factors play an important role that affects the risk specific diseases or sensitivity to therapeutic drugs in a conventionally defined racial group (Howard et al., 2019; Geneviève et al., 2020). Following those instances, prenatal PCB exposure in the Romani population from the same Slovak region showed an association with birth weight (Sonneborn et al., 2008a, b). The maternal PCB levels were linked with lower birth weight in Romani boys. The higher levels of PCBs in maternal blood sera may restrict the growth in boys, influenced by social factors related to ethnicity (Sonneborn et al., 2008a, b; Park et al., 2008). In our group of 45-month-old children, higher exposure to PCBs, DDE, DDT, and HCH in Romani children was associated with higher downregulation of selected genes (except *CYP2D6* and *ENTPD3*) (Fig. 4). In view of frequent congenital malformations, consanguinity, and high incidence of genetically conditioned diseases in the Roma population (Hajioff and McKee, 2000; Bartosovic 2016; Kalaydjieva et al., 2001), our results on gene expression were not surprising. In prior research on the genetic susceptibility of the Slovak Roma population, associations were observed such as the mutation W24X in the GJB2 gene (Minárik et al. 2012), mutations in NDRG1 and HK1 genes (Gabrikova et al., 2013), occurrence of pathogenic variants in the ACADS gene (Lisyová et al., 2018), incidence of Crigler-Najjar syndrome type I (Zmetáková et al., 2007), high incidence of primary congenital glaucoma (gene symbol GLC3) (Genčík et al., 1982; Plásilová et al. 1998), higher occurrence of MCAD deficit (Bzddúch 2006), and a frequent mutation of the phenylalanine hydroxylase gene (Kalanin et al., 1994). Moreover, the Slovak inhabitants of Roma ethnicity are considered a group with a higher risk of cardiovascular disease (Hujová et al. 2010), and others have observed heterogeneity in genetic profiles of the Slovak Romany (Gypsy) sub-ethnic groups (Siváková et al., 1994; Bernasovský et al., 1994). The lack of detailed information on the health indices of minority population (Roma here), demands the need for additional research on their health and cultural concerns (Zeman et al., 2003).

*ENTPD3* showed maximum downregulation (1.21-fold change) in our study that encrypts a plasma membrane-bound divalent cation-dependent E-type nucleotidase and is engaged in the control of extracellular concentrations of ATP by hydrolysis and additional nucleotides. Our result strongly corroborated and supports that the downregulation of the *ENTPD3* gene during the early developmental stage may lead to functional deficits and increased risks of diabetics or even cancer in later life (Li et al., 2019), although, as per our observation, the level of gene expression did not correlate with the other important exposure conditions (e.g., DDT, DDE, HCH) (data not shown).

There is sufficient evidence that PCB exposure is associated with disease development in children, whereas a study from 2012 equally recommends that additional prenatal OC exposures including HCH, DDE, and DDT have been related to being overweight at 6.5 years of age (Valvi et al., 2012). HCH isomers are also classified as potential human carcinogens and endocrine disruptors with established teratogenic, mutagenic, and genotoxic effects. They are rapidly absorbed from the gastrointestinal tract, and they crossed the placental barrier and are also transferred into breast milk. γ-HCH has the most acute neurotoxicity followed by α-HCH, whereas less β-HCH permeates the central nervous system (Berntssen et al., 2017). In our study, β-HCH was more prevalent in the population compared to the other two isomers (α and γ), yet we observed a linear association of γ-HCH with *MYC* and *CYP2D6* gene downregulation. Our previous study reported the prospective molecular effects of HCH exposure on a genomic level with possible molecular impairments and disease risks (Mitra et al., 2012). And finally, the effect of DDE and DDT has been extensively studied for their toxicity and carcinogenicity in animals and humans, and as endocrine disruptors (Harada et al., 2016). We also detected high amounts of DDE and DDT exposure in the study population. The level of exposure also correlated with downregulation of the *LEPR* gene, which is also corroborated with the earlier studies in the Slovak cohort (Ghosh et al., 2013; 2015).

In conclusion, our results suggest that environmental exposures to persistent organochlorine pollutants, especially PCBs, are associated with measurable effects on gene expression levels at the age of 45 months. In our Slovakian cohort, there are also consistent effects of male gender, Roma ethnicity, and higher BMI levels on gene expression patterns. Taken together, the results have implications for the future health of these children, for whom attentive surveillance is warranted for the future development of metabolic syndrome and other multi-systemic diseases including cancer. Efforts to mitigate such health effects of the legacy of environmental pollution must also be strengthened and extended to other regions and populations around the globe. The identification of any health disparities, i.e., the vulnerability of the Roma children in our study, must also be emphasized in such ongoing and future research, together with interventions to reduce the health consequences of such disparities. Finally, we emphasize the importance of biomarker-based approaches to future research on genetic susceptibility.

## Data Availability

Not applicable.
